# Endogenous auxin and its manipulation influence *in vitro* shoot organogenesis of citrus epicotyl explants

**DOI:** 10.1038/hortres.2017.71

**Published:** 2017-12-13

**Authors:** Wei Hu, Sabrina Fagundez, Lorenzo Katin-Grazzini, Yanjun Li, Wei Li, Yingnan Chen, Xiaomin Wang, Ziniu Deng, Shenxi Xie, Richard J McAvoy, Yi Li

**Affiliations:** 1 National Center for Citrus Improvement, Horticulture and Landscape College, Hunan Agricultural University, Changsha 410128, People’s Republic of China; 2 Department of Plant Science and Landscape Architecture, University of Connecticut, Storrs, CT 06269, USA; 3 Institute of Botany, Jiangsu Province and Chinese Academy of Sciences, 210014 Nanjing, People’s Republic of China

## Abstract

Endogenous auxin is an important regulator of *in vivo* organ development, but its role in *in vitro* organogenesis is unclear. It has been observed that the basal end of epicotyl cuttings of juvenile citrus seedlings produces fewer shoots than the apical end. Here, we report that elevated endogenous auxin levels in the basal end of citrus epicotyl cuttings are inhibitory for *in vitro* shoot organogenesis. Using transgenic citrus plants expressing an auxin-inducible *GUS* reporter gene, we have observed elevated levels of auxin at the basal end of stem cuttings that are mediated by polar auxin transport. Depleting endogenous auxin or blocking polar auxin transport enhances shoot organogenesis. An auxin transport inhibitor, N-1-naphthylphthalamic acid (NPA), can also enhance shoot organogenesis independent of its action on polar auxin transport. Finally, we demonstrate that the promotional effects of depleting endogenous auxin or blocking polar auxin transport on shoot organogenesis are cytokinin-dependent. Our study thus provides meaningful insights into possible roles of endogenous auxin and polar auxin transport, as well as auxin–cytokinin interactions, in *in vitro* shoot organogenesis. Meanwhile, our results may also provide practical strategies for improving *in vitro* shoot organogenesis for citrus and many other plant species.

## Introduction

Auxin has a crucial role in the regulation of the spatial and temporal aspects of plant growth and development. Intercellular transport of auxin from shoot apexes to more basal plant tissues affects a wide array of growth and developmental processes, including cell elongation and cell division. Auxin contributes to plant organogenesis; for instance, auxin is important for determining cell division plane orientation prior to lateral root initiation.^1^ In addition, epidermis-localized auxin triggers leaf and flower initiation.^2,3^ In addition, auxin has been reported to be involved in the formation of meristems.^4^ Further, it has been proposed that shoot apical meristem development depends on auxin, not only through the regulation of auxin synthesis and transport but also through the creation of auxin gradients and, therefore, local auxin maxima.^5^ Thus, auxin is one of the most important classes of plant hormones for *in vivo* organ development. However, it is not well understood whether or how endogenous auxin influences shoot organogenesis under tissue culture conditions.

The presence of auxin in tissue culture medium leads to pericycle cell division, from which founder cells emerge.^6–8^ In an auxin-rich environment, these founder cells will undergo further cell division to form genetically distinct calli.^9–11^ At this stage, organogenesis will occur with shoots and/or roots developing from the calli.^12^ If calli are transferred to high-cytokinin and low-auxin medium, then shoot organogenesis will begin. However, on high-auxin but low-cytokinin medium, root organogenesis will occur with shoot organogenesis being inhibited.^13^ Although some auxin response factors, such as *ARF3*, may repress cytokinin biosynthesis,^14^ the auxin and cytokinin interactions involved in *in vitro* organogenesis are not well understood.^15,16^

Some citrus species are recalcitrant to shoot organogenesis, especially following genetic transformation. Donmez *et al.*^17^ remarked, ‘citrus transformation efficiency is generally low and protocols are dependent on species or even cultivars.’ Although reasonably high regeneration efficiencies have been achieved for some citrus cultivars using juvenile materials, many others, such as lemon (*Citrus limon* L.), continue to trouble researchers.^18,19^ We have recently demonstrated that overexpression of cytokinin-related genes such as *kn1* or *ipt* can improve shoot organogenesis for a number of citrus cultivars, including lemon.^20^ However, it is not known whether endogenous auxin has a role in shoot organogenesis in citrus explants.

In this study, we explored the impact of endogenous auxin on shoot organogenesis in two citrus cultivars: ‘Carrizo’ citrange [*Citrus sinensis* (L.) Osbeck×*Poncirus trifoliata* (L.) Raf.] and ‘Eureka’ lemon [*Citrus limon* (L.) Burm. f.]. We found that the accumulation of endogenous auxin due to polar auxin transport inhibited shoot organogenesis at the basal end of epicotyl segment explants. We used N-1-naphthylphthalamic acid (NPA) to inhibit polar auxin transport or washed explants with liquid to remove endogenous auxin, and found that this was sufficient to promote shoot formation. Finally, we demonstrated that the promotional effect of reduced endogenous auxin on shoot organogenesis was cytokinin-dependent.

## Materials and methods

### Plant materials

‘Carrizo’ citrange [*Citrus sinensis* (L.) Osbeck×*Poncirus trifoliata* (L.) Raf.] seeds were bought from Tree Source Citrus Nursery (504 N Kaweah Ave., Exeter, CA 93221 USA), and ‘Eureka’ lemon [*Citrus. limon* (L.) Burm. f.] seeds were bought from Pearson Ranch California Oranges (1018W. Teapot Dome Ave., Porterville, CA 93257 Usa). Outer seed coats were removed manually, and seeds were treated with 75% alcohol for 1 min., followed by 1% sodium hypochlorite for 20 min., and then rinsed four times with sterile distilled water. Internal seed coats were removed under sterile conditions, and seeds were cultured *in vitro* on MS medium ^21^ with 30 g L^−1^sucrose and 7 g L^−1^ agar at pH 5.7. Seedlings were stored in the dark for 15 days before being transferred to light conditions (~60 μmol m^-2^ s^-1^ PPFD) with a 16-h photoperiod. Internodal stem segments from young branches of greenhouse-grown *DR5::uidA* ‘Carrizo’ citrange plants were used as explants for GUS staining experiments.

### Shoot regeneration

The epicotyls of 30-day-old citrus seedlings were sectioned into 1-cm internodal segments using two different cutting styles: (1) A transecting (‘blunt’) cut at the apical end of the explant and a slant cut at the basal end, or (2) A transecting (‘blunt’) cut at both ends of the explant. The explants were then cultured on shoot regeneration medium (SRM), which was composed of MS medium, 30 g L^−1^ of sucrose, 7 g L^-1^ of agar, 0–3 mg L^-1^ benzylaminopurine (BAP) and 0–20 mg L^-1^ NPA, depending on treatment type. All SRM contained 3 mg L^-1^ BAP and 0 mg L^-1^ NPA unless stated otherwise. The explant tissues were cultured under light conditions (60 μmol m^-2^ s^-1^) at a 16-h photoperiod (26±2 °C) and were transferred onto fresh SRM every 3 weeks.

### GUS histochemical assays

*DR5::uidA* internodal stem segments (~1 cm in length) from the greenhouse were used for histochemical GUS staining. Histochemical assays of GUS activity were carried out at 37 °C for 16 h in a solution consisting of 100 mM potassium phosphate buffer with pH 7, 10 mM Na_2_EDTA, 0.5 mM K_3_Fe(CN)_6_, 0.5 mM K_4_Fe(CN)_6_, 0.1% Triton X-100 and 1 g L^-1^ X-gluc (5-bromo-4-chloro-3-indolyl-β-D-glucuronic acid). The plant tissues were destained in ethanol to gradually remove chlorophyll and other pigments before photographs were taken.^22^

### Auxin treatment and incubation

Auxin treatment: Blunt-cut *DR5::uidA* internodal stem explants were incubated in a 10 mM potassium phosphate buffer (pH 6) containing 50 μg mL^-1^ chloramphenicol with or without auxin (IAA). The tissues were incubated in the dark with gentle agitation on a rotary shaker at room temperature for 24 h before being analyzed for GUS activity.

Incubation to remove endogenous auxin: Blunt-cut *DR5::uidA* internodal stem explants were incubated in liquid MS medium in the dark to remove their endogenous auxin. The tissues were incubated in the dark with gentle agitation on a rotary shaker at room temperature for 24 h before being analyzed for GUS activity.

### Statistical analyses

Statistical analysis was performed using h SPSS 21.0 software (IBM, New York, NY, USA). The statistical significance of the experiment was determined either by the two-tailed Student’s *t*-test with *α*=0.05 (comparison between two treatments) or by the single-factor ANOVA (comparison among three or more treatments).

## Results

### Polar auxin transport-mediated increases in endogenous auxin content inhibit *in vitro* shoot organogenesis of citrus epicotyl explants

**Figure 1:**
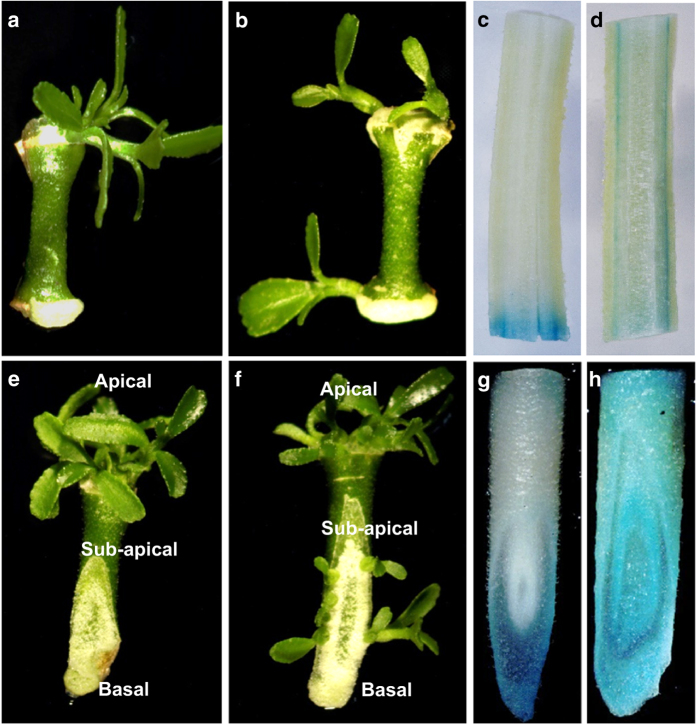
Elevated endogenous auxin level in the basal end of explants inhibits shoot organogenesis of ‘Carrizo’ citrange stem segments. In all pictures, explants are arranged with the apical end facing up and the basal end facing down. (**a**) An internodal stem explant with a blunt basal end formed shoot primordia on the apical end, but shoot initiation was repressed on the basal end. (**b**) When N-1-naphthylphthalamic acid (NPA) was applied to blunt-cut internodal stem explants, shoot primordia formed on both the apical and basal ends of the explant. (**c**) GUS-stained *DR5::uidA* blunt-cut internodal segments show a blue color at the basal end, which is indicative of an elevated auxin level. (**d**) When NPA was applied to *DR5::uidA* blunt-cut internodal segments, a blue color was evenly distributed across the explant, indicating polar auxin transport was inhibited. (**e**) An internodal stem explant with a slanted end had shoots initiated from the upper region of the cut (sub-apical). (**f**) When NPA was applied to slant-cut internodal stem explants, shoot primordia formed at both the apical and basal ends of the cut. (**g**) GUS-stained *DR5::uidA* slant-cut internodal segments show a blue color at the basal end of the cut, indicating elevated auxin in these cells due to polar auxin transport. (**h**) When NPA was applied to *DR5::uidA* slant-cut internodal segments, a blue color was evenly distributed across the explant, indicating that polar auxin transport was inhibited.

Figures 1a and e show that shoot organogenesis was biased towards the apical end of the explants for both blunt and slant cuts of ‘Carrizo’ citrange. When slant cuts were used, shoot initiation occurred at the sub-apical region of the basal end (the apical end of the cut). To examine effects of endogenous auxin and polar auxin transport on shoot initiation, especially regarding the repression of shoot initiation at the basal end, we examined *GUS* expression in blunt-cut and slant-cut transgenic *DR5::uidA* stem segments. *DR5* is a synthetic auxin-responsive promoter;^23^ therefore, *GUS* expression could be indicative of relative auxin levels and localizations in these explants. We detected higher GUS activity, and presumably elevated auxin levels, in the basal end of explants (Figures 1c and g). *GUS* gene activation, indicative of an elevated auxin level, provides an explanation of why shoot organogenesis was inhibited in the basal end of explants.

To obtain more evidence for the involvement of endogenous auxin and polar auxin transport on the inhibition of *in vitro* shoot organogenesis, we treated blunt-cut and slant-cut ‘Carrizo’ citrange explants with NPA, a polar auxin transport inhibitor. Figures 1b and f show that shoot initiation also occurred in the basal end of epicotyl segments if NPA was applied. NPA blocked auxin accumulation in the basal end of explants, as indicated by the equal distribution of GUS activity (expression of the *DR5::uidA* gene) along the entire explant (Figures 1d and h). These results provide evidence that the elevated auxin level observed in the basal end of the ‘Carrizo’ citrange epicotyl explants caused by polar auxin transport inhibits shoot organogenesis.

**Figure 2:**
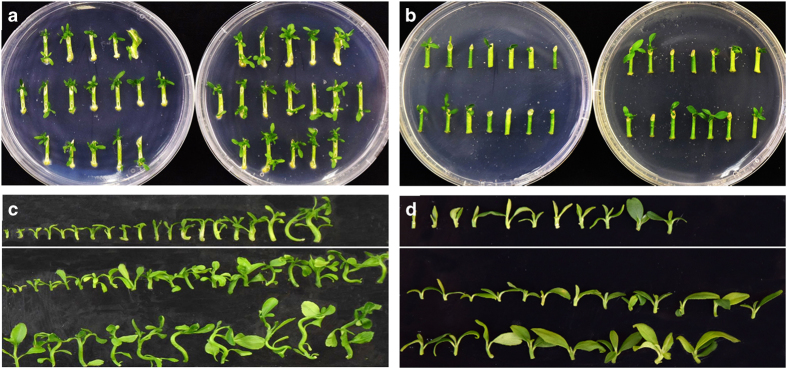
Elevated auxin levels due to polar auxin transport inhibit shoot organogenesis at the basal end of citrus stem segments. In all pictures, explants are arranged with the apical end facing up and the basal end facing down. (**a**) After 15 days, untreated ‘Carrizo’ citrange explants (left) had inhibited shoot development at the basal end, whereas explants treated with N-1-naphthylphthalamic acid (NPA) (right) experienced shoot development at both the apical and basal ends of the explant. (**b**) After 15 days, untreated ‘Eureka’ lemon explants (left) experienced reduced shoot development compared to explants treated with NPA (right). (**c**) Shoots produced from five untreated ‘Carrizo’ citrange explants (above the white line) were fewer in number and smaller in size compared to shoots from five explants treated with NPA (below the white line). (**d**) Five of the NPA-treated ‘Eureka’ lemon explants produced more and larger-sized shoots (below the white line) than the untreated controls (above the while line).

We treated ‘Carrizo’ citrange and ‘Eureka’ lemon (*Citrus limon* L.) epicotyl segments with NPA and examined shoot organogenesis of the explants. We observed that NPA treatment significantly enhanced shoot organogenesis for both ‘Carrizo’ citrange (increased from 3.93 to 7.48 shoots per explant) and ‘Eureka’ lemon (increased from 0.79 to 1.97 shoots per explant) (Figures 2a–d; Table 1). NPA treatment significantly enhanced shoot organogenesis at the basal end of these explants, with increases from 1.55 to 3.61 shoots per explant for ‘Carrizo’ citrange and from 0 to 0.80 shoots per explant for ‘Eureka’ lemon. NPA also enhanced shoot growth (likely due to the earlier initiation of buds) for both ‘Carrizo’ citrange and ‘Eureka’ lemon (Figures 2c and d). These results demonstrate that when polar auxin transport is inhibited by NPA, shoot organogenesis from epicotyl segments is enhanced, providing additional evidence that elevated endogenous auxin is inhibitory for *in vitro* shoot organogenesis.

### Additional evidence supports endogenous auxin-mediated inhibition of shoot organogenesis

**Figure 3:**
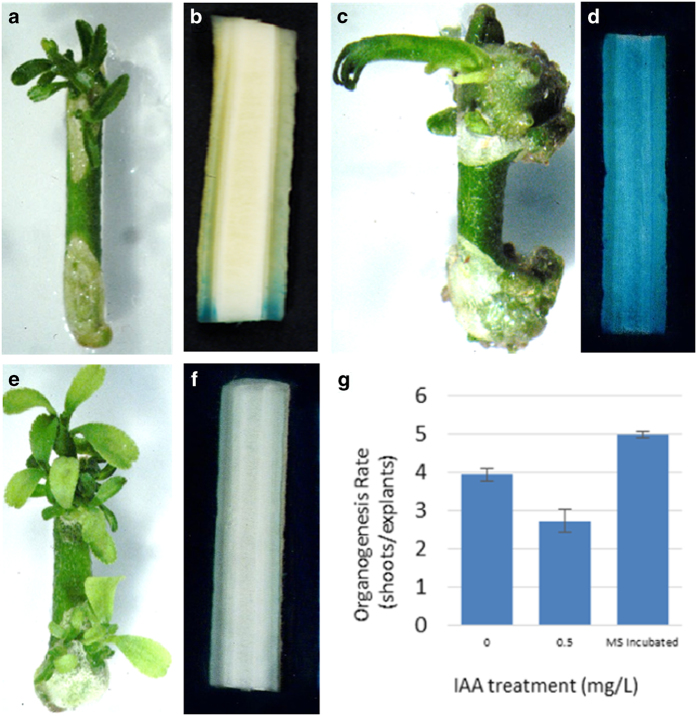
Auxin concentrations are negatively correlated with shoot organogenesis on ‘Carrizo’ citrange stem segments. In all pictures, explants are arranged with the apical end facing up and the basal end facing down. (**a**) Untreated explants produced calli at both ends and developed shoots from the apical end. (**b**) GUS-stained *DR5::uidA* explants had higher GUS expression at the basal end due to polar auxin transport. (**c**) Explants treated with 0.5 mg L^-1^ exogenous IAA had enhanced callus production from both ends and had reduced shoot organogenesis. (**d**) GUS-stained *DR5::uidA* explants had elevated GUS activity in the entire explant because of exogenous application of auxin (0.5 mg L^-1^ indole-3-acetic acid (IAA)). (**e**) Explants incubated with MS medium (wash) for 2 h to deplete endogenous auxin produced shoots from both the apical and basal ends. (**f**) GUS-stained *DR5::uidA* explants had no detectable GUS activity after the wash. (**g**) Shoot organogenesis was negatively correlated with auxin concentration: ‘0’—no auxin was added to the solid MS medium; ‘0.5’—0.5 mg L^-1^ IAA was added to the solid medium; MS incubated—explants were washed with MS liquid medium for 2 h to deplete endogenous auxin.

To further explore the role that auxin has in shoot organogenesis, we treated ‘Carrizo’ citrange epicotyl segments with 0.5 mg L^-1^ exogenous indole-3-acetic acid (IAA). These IAA-treated explants had reduced shoot organogenesis, as well as increased callus formation (Figure 3c), compared to untreated explants (Figure 3a). In addition, we incubated ‘Carrizo’ citrange epicotyl segments in hormone-free MS liquid medium with moderate agitation (explant wash) for 2 h to deplete endogenous auxin. The wash significantly enhanced shoot organogenesis from the basal end of explants from 1.35 to 2.95 shoots per explant (Figure 3e; Table 2). With the application of NPA, we also observed enhanced shoot organogenesis, with 2.81 shoots produced per explant. An increase to 3.67 shoots per explant was observed if the use of NPA was combined with the explant wash (Table 2). These results also demonstrate that NPA can enhance shoot organogenesis independent of its effect on polar auxin transport.

We used *DR5::uidA* stem segments to monitor changes in endogenous auxin levels after the explant wash. We observed significant amounts of GUS activity in non-washed explants but undetectable GUS activity in washed explants (Figure 3f), demonstrating the drastic reduction of endogenous auxin levels in the washed explants. Our results show that the explant wash is effective for removing endogenous auxin from explants. Table 2 shows that shoot organogenesis in the basal end of explants was negatively correlated with auxin content, with an increase in shoot organogenesis from 1.35 shoots (unwashed) to 2.95 shoots (washed) per explant. In contrast, treatment with exogenous IAA led to a significant reduction in the total number of shoots produced from both apical and basal ends of explants (reduced from 5.12 shoots to 2.71 shoots per explant) (Figure 3g). These results demonstrate that relatively high auxin concentrations suppress shoot organogenesis from the basal end of epicotyl segments and suppress total numbers of shoots from these explants.

### Cytokinin is required for the NPA promotional effect on *in vitro* shoot organogenesis

To determine the impact of polar auxin transport on *in vitro* organogenesis with and without exogenously applied cytokinin, we treated ‘Carrizo’ citrange epicotyl segments with NPA and various concentrations of 6-benzylaminopurine (BAP) and tracked shoot organogenesis. Without exogenous cytokinin, 1.11 shoots were produced per explant if no NPA was used, and 1.14 shoots were produced per explant if NPA was used (Table 3), indicating that NPA has no effect if no cytokinin is used. If 3 mg L^-1^ BAP was used, we observed a total of 3.93 shoots produced per explant when no NPA was used. Under the same concentration of BAP, the application of NPA-enhanced shoot organogenesis to a total of 6.47 shoots produced per explant, demonstrating NPA can have a promotional effect if cytokinin is present. We also observed that NPA’s ability to alleviate auxin-mediated repression of shoot organogenesis at the basal end of epicotyl segments was dependent on the presence of cytokinin. At the same NPA concentration (20 mg L^-1^), the shoot organogenesis rate was 1.03 shoots per basal end of explants if 0.5 mg L^-1^ BAP was used and 2.61 shoots per basal end of explants if 3 mg L^-1^ BAP was used (Table 3).

## Discussion

In this study, we have demonstrated that elevated levels of endogenous auxin in the basal end of epicotyl segments inhibit shoot organogenesis. We have also shown that the elevated auxin levels at the basal end of epicotyl explants are caused by polar auxin transport. We further show that reducing endogenous auxin content in explants by washing or blocking polar auxin transport with an auxin transport inhibitor (NPA) promotes shoot organogenesis. Our results indicate that NPA also enhances shoot organogenesis independent of its involvement in auxin transport. Finally, we have demonstrated that the promotional effect of NPA on shoot organogenesis is cytokinin-dependent. Our findings thus provide some insights into the involvement of auxin polar transport, auxin and auxin’s interaction with cytokinin in *in vitro* shoot organogenesis, as well as possible strategies for improving the efficiency of shoot organogenesis for citrus and other plant species.

We have shown that polar auxin transport is responsible for the smaller number of shoots regenerated from the basal end of explants. Auxin is generally synthesized in young leaves and shoot apical meristems, after which it is transported basipetally in the stem.^24^ The distribution of endogenous auxin, controlled mainly by the activity of polar auxin transporters, is known to be vital during organogenesis and various tropisms.^25,26^ During early embryogenesis, auxin has a major role in the determination of important cell lineages, such as the apical cell.^27^ During plant development, auxin is involved in leaf, flower and root development, as well as the maintenance of meristems, mostly through the creation of auxin gradients, which is facilitated by polar auxin transport.^4,12,28–30^ Our study has demonstrated that endogenous auxin in the basal end of explants is too high for shoot organogenesis and thus produces inhibitory effects. Further, we provide experimental evidence that the higher auxin level in the basal end of epicotyl or stem explants is caused by polar auxin transport.

The higher shoot regeneration rates observed in the apical end of epicotyl explants are also due to polar auxin transport. Polar auxin transport reduces auxin content in the apical end of epicotyl explants. Previously, it was shown that the application of exogenous auxin can have a negative effect on shoot regeneration in tissue culture.^31,32^ For example, cucumber explants exposed to auxin-rich media preferentially produced calli while experiencing a reduction in direct shoot regeneration.^33^ Like citrus, some other woody plant taxa are also recalcitrant to shoot organogenesis in tissue culture.^34^ In light of our recent findings, it is possible that this recalcitrance may be partially due to relatively high concentrations of endogenous auxin. Neutralizing the negative effects of endogenous auxin, either through its removal (washing the explants with liquid) or through the inhibition of polar auxin transport, may enhance shoot organogenesis in many plant species.

The effects of auxin and cytokinin on *in vitro* organogenesis are well known.^15,16,21,35,36^ When explants are placed on a medium that has a high auxin-to-cytokinin ratio, they will develop calli.^10,11^ If they are on a medium with a high cytokinin-to-auxin ratio, they will develop buds/shoots.^37–39^ Our results suggest that cytokinin is the primary factor for shoot organogenesis, and auxin inhibits the effect of cytokinin in shoot organogenesis. We have observed that NPA has a promotional effect on shoot organogenesis from citrus epicotyl explants only in the presence of exogenous cytokinin. Without added cytokinin, NPA has little effect on *in vitro* organogenesis.

We have demonstrated that either the use of auxin transport inhibitors or the physical depletion of endogenous auxin from explants may improve *in vitro* shoot organogenesis for some recalcitrant plant species. Our results are consistent with the findings of previous studies using NPA to increase shoot organogenesis in tobacco ^40,41^ or PCIB, an anti-auxin, to enhance shoot regeneration in guar (*Cyamopsis tetragonoloba*).^42^ In addition, we have observed that NPA enhances shoot organogenesis, even after endogenous auxin is washed off of the explants via incubation in hormone-free liquid medium, which suggests that NPA has additional activity independent of blocking auxin transport.

## Figures and Tables

**Table 1:** NPA enhances shoot organogenesis in citrus

*Cultivar*	*NPA (20 mg L*^*-1*^)	*Apical-end organogenesis rate*^a^	*Basal-end organogenesis rate*^a^	*Total organogenesis rate*^a^
‘Carrizo’ citrange	−	2.38±0.16	1.55±0.02	3.93±0.17
	**+**	3.87±0.19*	3.61±0.16*	7.48±0.23*
‘Eureka’ lemon	−	0.58±0.10	0	0.79±0.15
	**+**	1.17±0.17*	0.80	1.97±0.23*

a Shoot organogenesis rate was calculated by dividing the total shoot number by the total explant number.

Asterisk represents a significant difference when compared to the same cultivar with no NPA treatment using two-tailed Student's t test (*P*≤0.05).

**Table 2:** Wash-mediated depletion of endogenous auxin enhances shoot organogenesis

*NPA (20 mg L*^*-1*^)	*Treatment*	*Apical-end organogenesis*+	*Basal end organogenesis*+
−	No wash	2.97±0.18^b^	1.35±0.22^a^
**+**	No wash	4.25±0.25^d^	2.81±0.27^b^
−	Wash++	3.56±0.23^c^	2.95±0.18^b^
**+**	Wash++	4.87±0.41^e^	3.67±0.31^c^

+ Shoot organogenesis rate was calculated by dividing the total shoot number by the total explant number.

++ Explants were incubated in liquid MS medium with agitation for 2 h to wash endogenous auxin from the tissues.

Values followed by the different letters are significantly different at *P*<0.05 (ANOVA; LSD).

**Table 3:** NPA promotes cytokinin-mediated shoot organogenesis in ‘Carrizo’ citrange+

*Cytokinin (BAP) (mg L*^*-1*^)	*NPA (20 mg L*^*-*1^)	*Apical-end organogenesis rate*++	*Basal-end organogenesis rate*++	*Total organogenesis rate*++
0	−	1.04±0.04^c^	0.07±0.07^a^	1.11±0.11^c^
0	**+**	1.14±0.10^c^	0	1.14±0.10^c^
0.5	−	2.24±0.12^e^	0.65±0.30^b^	2.89±0.41^f^
0.5	**+**	2.74±0.21^f^	1.03±0.03^c^	3.76±0.18^g^
1	−	2.15±0.15^e^	1.15±0.15^c^	3.30±0.01^f,g^
1	**+**	3.03±0.50^f^	1.68±0.03^d^	4.76±0.48^h^
3	−	2.38±0.16^e^	1.55±0.32^d^	3.93±0.17^g^
3	**+**	3.87±0.19^g^	2.61±0.05^f^	6.47±0.18^i^

+ NPA is an auxin transport inhibitor. The fact that NPA’s promotional effects on shoot organogenesis are cytokinin-dependent indicates auxin represses the cytokinin-mediated shoot organogenesis.

++ Organogenesis rate was calculated by dividing the total shoot number by the total explant number.

Values followed by the different letters are significantly different at *P*<0.05 (ANOVA; LSD).
